# Supplementary figures and images for: Re-programming of Pseudomonas syringae pv. actinidiae gene expression during early stages of infection of kiwifruit
Source: BMC Genomics. 2018 Nov 15;19:822. doi: 10.1186/s12864-018-5197-5 (PMC6238374; doi:10.1186/s12864-018-5197-5)

## Slide 1
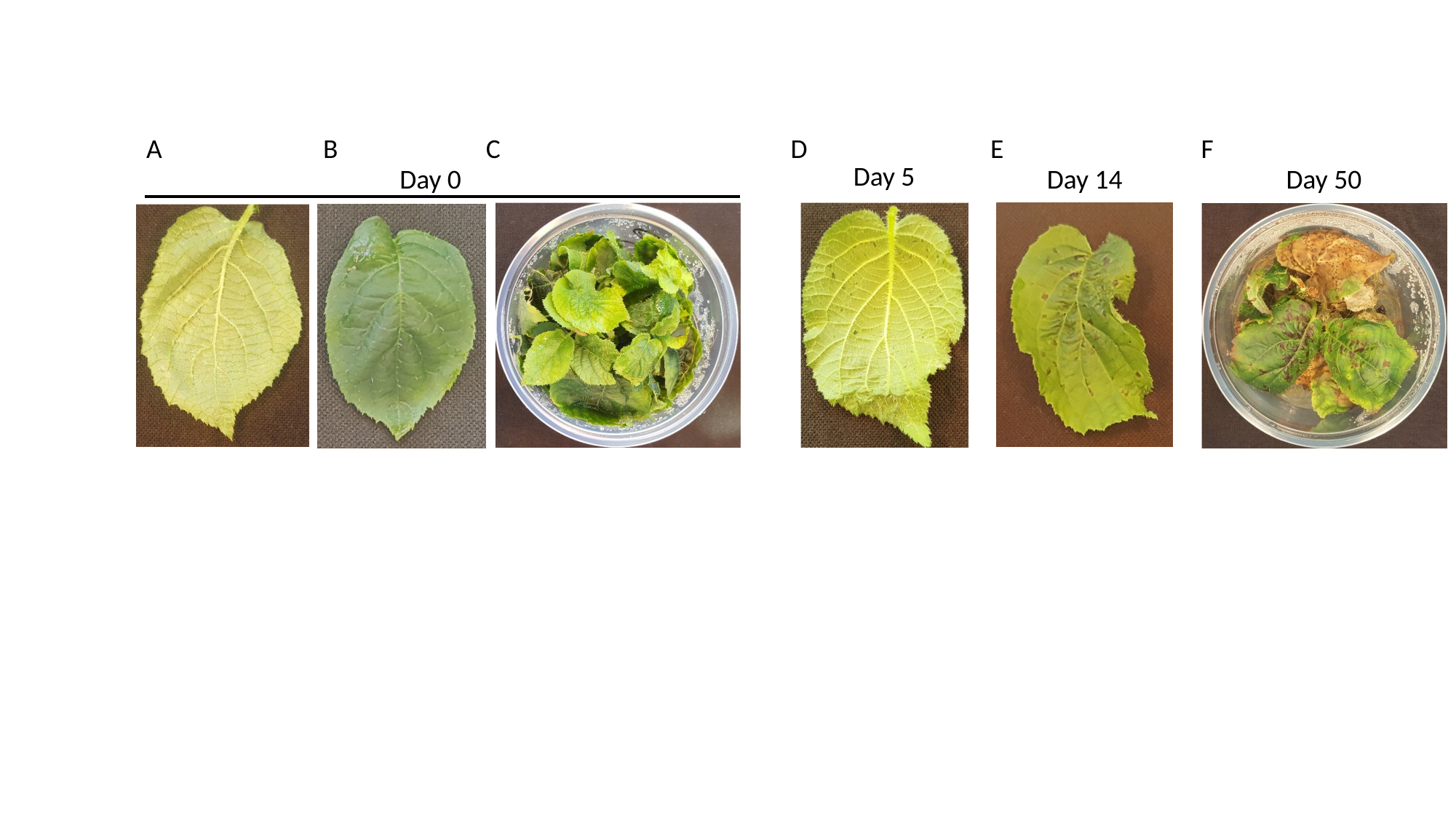

A	 B		 C		 D	 E F
Day 5
Day 0
Day 14
Day 50

Supplement: Supplementary file 1 — Images illustrating the time course of symptom development of kiwifruit plantlets infected with Psa. (A) Abaxial side of leaf at day 0; (B) Adaxial side of leaf at day (0); (C) Pottle containing plantlets at day 0; (D) Abaxial side of leaf five days post inoculation (DPI) with water soaked lesions appearing; (E) Adaxial side of leaf 14 DPI with necrotic lesions present; (F) Plantlets 50 DPI. (PPTX 10001 kb) [file 12864_2018_5197_MOESM1_ESM.pptx]

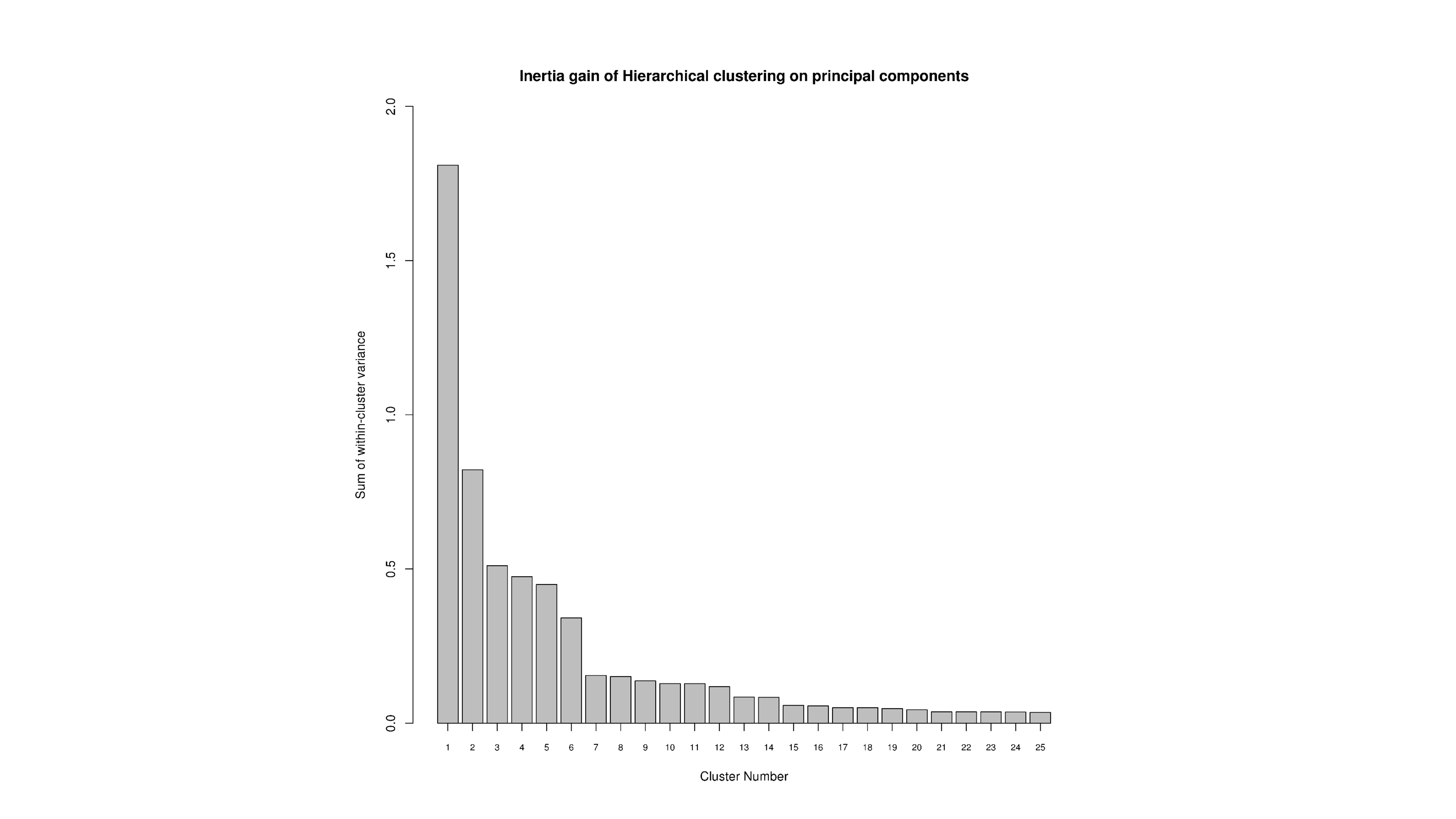

Supplement: Supplementary file 13 — A bar plot of the inertia gain using the sum of the within-group variance with increasing cluster number (x-axis) produced using Hierarchical clustering on principal components. (JPG 742 kb) [file 12864_2018_5197_MOESM13_ESM.jpg]
