# Supplementary material for: Re-programming of Pseudomonas syringae pv. actinidiae gene expression during early stages of infection of kiwifruit
Source: BMC Genomics. 2018 Nov 15;19:822. doi: 10.1186/s12864-018-5197-5 (PMC6238374; doi:10.1186/s12864-018-5197-5)
Supplement: Supplementary file 3 — Early induced genes ranked by the ratio of expression at three hours post infection (HPI) compared with in vitro (cutoff 5-fold). (DOCX 20 kb) [file 12864_2018_5197_MOESM3_ESM.docx]

Additional file 3. Early induced genes ranked by the ratio of expression at 3 hours post inoculation (HPI) compared with *in vitro* (cutoff 5-fold).

| Gene ID | Gene Annotation | 3 HPI/ *in vitro* | *P* value |
| --- | --- | --- | --- |
| IYO_011995 | phosphate ABC transporter substrate-binding protein | 148.8 | 4.60E-41 |
| IYO_012000 | phosphate ABC transporter permease | 43.7 | 3.31E-29 |
| IYO_019585 | thioredoxin | 31.4 | 6.68E-31 |
| IYO_012010 | phosphate ABC transporter ATP-binding protein | 30.8 | 1.50E-33 |
| IYO_018790 | magnesium transporter CorA | 30.8 | 8.40E-24 |
| IYO_027385 | ABC transporter substrate-binding protein | 29.5 | 1.15E-19 |
| IYO_027390 | GntR family transcriptional regulator | 25.0 | 2.15E-13 |
| IYO_015395 | hypothetical protein | 23.9 | 1.38E-10 |
| IYO_006115 | amino acid ABC transporter substrate-binding protein | 23.3 | 8.31E-37 |
| IYO_027380 | ABC transporter permease | 21.5 | 1.65E-13 |
| IYO_028665 | phosphate-binding protein | 20.4 | 5.18E-52 |
| IYO_018545 | acid phosphatase | 19.5 | 5.16E-26 |
| IYO_000970 | ammonia channel protein | 18.7 | 1.03E-26 |
| IYO_006555 | chemotaxis protein | 17.4 | 3.36E-61 |
| IYO_021410 | short-chain dehydrogenase | 17.2 | 4.98E-09 |
| IYO_013555 | type VI secretion effector protein (Hcp) | 16.3 | 1.59E-20 |
| IYO_013560 | EvpB family type VI secretion protein | 15.9 | 9.21E-28 |
| IYO_028645 | transcriptional regulator PhoU | 14.3 | 7.85E-27 |
| IYO_002185 | peptidase M19 | 13.6 | 7.60E-17 |
| IYO_010675 | phosphatase | 12.6 | 7.44E-13 |
| IYO_021050 | ABC transporter substrate-binding protein | 12.5 | 4.36E-34 |
| IYO_013550 | Type VI secretion protein | 12.2 | 2.07E-16 |
| IYO_013565 | type VI secretion protein | 11.7 | 2.77E-16 |
| IYO_001685 | MFS transporter | 11.6 | 1.44E-39 |
| IYO_021420 | polysaccharide deacetylase | 10.9 | 4.26E-14 |
| IYO_020035 | ABC transporter substrate-binding protein | 10.8 | 5.31E-20 |
| IYO_027365 | metallophosphatase | 10.4 | 2.18E-08 |
| IYO_014740 | sugar ABC transporter substrate-binding protein | 10.2 | 3.60E-29 |
| IYO_002190 | hydrocarbon binding protein | 9.8 | 3.80E-06 |
| IYO_020310 | hypothetical protein | 9.7 | 4.61E-06 |
| IYO_013545 | type VI secretion system protein ImpG | 9.5 | 1.15E-16 |
| IYO_006130 | glutamine ABC transporter ATP-binding protein | 9.4 | 4.49E-20 |
| IYO_028615 | transcriptional regulator PhoB | 9.2 | 5.26E-17 |
| IYO_004270 | chemotaxis protein | 9.1 | 5.83E-19 |
| IYO_001975 | nitrogen regulation protein NR(I) | 9.1 | 2.23E-18 |
| IYO_002205 | electron transfer flavoprotein subunit alpha | 8.8 | 4.35E-05 |
| IYO_025185 | urease accessory protein UreG | 8.7 | 2.64E-07 |
| IYO_021405 | 3-oxoacyl-ACP reductase | 8.5 | 3.81E-04 |
| IYO_026920 | ABC transporter permease | 8.5 | 1.68E-06 |
| IYO_006120 | amino acid ABC transporter permease | 7.5 | 4.63E-25 |
| IYO_002210 | electron transfer flavoprotein subunit beta | 7.4 | 1.27E-02 |
| IYO_027370 | iron ABC transporter substrate-binding protein | 7.3 | 7.54E-10 |
| IYO_027375 | ABC transporter permease | 6.8 | 1.39E-03 |
| IYO_018365 | MFS transporter | 6.6 | 7.33E-05 |
| IYO_014745 | xylose isomerase | 6.3 | 3.08E-11 |
| IYO_018070 | ATPase | 6.2 | 9.96E-03 |
| IYO_006260 | acetyltransferase | 6.0 | 7.07E-06 |
| IYO_027495 | sarcosine oxidase subunit alpha | 5.9 | 5.62E-14 |
| IYO_001120 | hypothetical protein | 5.8 | 1.04E-04 |
| IYO_026150 | acyl carrier protein | 5.6 | 1.92E-01 |
| IYO_004585 | branched-chain amino acid ABC transporter substrate-binding protein | 5.6 | 4.05E-16 |
| IYO_009210 | quercetin 2,3-dioxygenase | 5.5 | 4.42E-03 |
| IYO_014735 | xylose transporter | 5.5 | 9.61E-09 |
| IYO_025180 | urease accessory protein UreF | 5.5 | 7.13E-04 |
| IYO_021415 | MFS transporter | 5.2 | 7.97E-05 |
