# Supplementary material for: Re-programming of Pseudomonas syringae pv. actinidiae gene expression during early stages of infection of kiwifruit
Source: BMC Genomics. 2018 Nov 15;19:822. doi: 10.1186/s12864-018-5197-5 (PMC6238374; doi:10.1186/s12864-018-5197-5)
Supplement: Supplementary file 4 — Genes most highly upregulated in the mid phase of the infection time course (3–24 h post infection, (HPI)). Genes are ranked on the ratio of maximum Reads Per Kilobase per Million over that time period compared with in vitro expression (cutoff 5-fold). (DOCX 24 kb) [file 12864_2018_5197_MOESM4_ESM.docx]

Additional file 4. Genes most highly upregulated in the mid phase of the infection time course (3-24 hours post inoculation). Genes are ranked on the ratio of maximum Reads per Kilobase per Million reads over that time period to *in vitro* expression (cutoff 5-fold).

| Gene ID | Gene Annotation | Ratio | *P*-value |
| --- | --- | --- | --- |
| IYO_006750 | type III secretion protein HrpW | 257.7 | 4.4E-64 |
| IYO_022020 | hemolysin | 176.9 | 9.7E-44 |
| IYO_006820 | type III secretion protein | 118.8 | 2E-88 |
| IYO_006755 | Shc Hop M1 (disrupted) | 104.0 | 1.2E-48 |
| IYO_006865 | type III secretion system protein | 77.2 | 2.7E-35 |
| IYO_006790 | HrpA1 | 77.0 | 1.7E-72 |
| IYO_004052 | HopS2 | 76.4 | 1E-37 |
| IYO_006825 | type III secretion protein | 67.5 | 7.8E-44 |
| IYO_006875 | type III secretion protein | 62.6 | 2.8E-28 |
| IYO_006880 | type III secretion protein | 54.0 | 1.8E-49 |
| IYO_006795 | type III secretion protein HrpZ | 52.4 | 3.5E-38 |
| IYO_012110 | Ais protein | 52.2 | 6.6E-21 |
| IYO_006905 | RNA polymerase sigma factor HrpL | 50.3 | 6E-38 |
| IYO_022025 | glycerol acyltransferase | 47.4 | 4E-16 |
| IYO_002060 | IAA lysine ligase | 46.4 | 6.2E-20 |
| IYO_004050 | type III chaperone ShcO1 | 44.3 | 8.2E-37 |
| IYO_006830 | secretin | 44.1 | 1E-37 |
| IYO_028770 | LysR family transcriptional regulator | 44.1 | 4.7E-25 |
| IYO_003325 | copper resistance protein CopZ | 41.4 | 8.7E-27 |
| IYO_006910 | type III effector HrpK | 39.6 | 3.5E-34 |
| IYO_012005 | phosphate ABC transporter permease | 37.7 | 3.1E-32 |
| IYO_028960 | sulfonate ABC transporter permease | 35.7 | 1.4E-12 |
| IYO_006420 | chemotaxis protein | 35.7 | 4.3E-25 |
| IYO_014395 | lytic transglycosylase | 31.2 | 4.2E-24 |
| IYO_014235 | hypothetical protein | 30.4 | 4.3E-21 |
| IYO_006890 | type III secretion protein | 30.4 | 9.3E-29 |
| IYO_006860 | type III secretion system protein SsaR | 30.2 | 5E-28 |
| IYO_006870 | type III secretion system protein | 29.3 | 1.4E-18 |
| IYO_006815 | type III secretion protein | 29.0 | 8.1E-30 |
| IYO_028955 | nitrate ABC transporter ATP-binding protein | 28.3 | 4.5E-08 |
| IYO_005735 | glycoside hydrolase | 25.8 | 1.2E-24 |
| IYO_006900 | type III secretion protein HrpJ | 25.2 | 3.3E-29 |
| IYO_003725 | type III chaperone protein ShcF | 23.8 | 1.1E-10 |
| IYO_006800 | type III secretion protein | 23.3 | 9.7E-29 |
| IYO_020425 | AvrPto5 | 23.3 | 3.3E-32 |
| IYO_006810 | type III secretion protein | 22.4 | 1.4E-30 |
| IYO_006805 | type III secretion protein | 22.1 | 1.6E-51 |
| IYO_006885 | ATP synthase | 21.3 | 1.6E-21 |
| IYO_006855 | type III secretory protein EscS | 20.8 | 1.3E-06 |
| IYO_006765 | type III chaperone ShcE | 18.8 | 5.1E-18 |
| IYO_006760 | HopM1 | 18.2 | 8.6E-32 |
| IYO_003570 | avrD1 | 18.0 | 4.8E-11 |
| IYO_014245 | membrane protein | 17.9 | 0.01651 |
| IYO_029290 | type III chaperone protein ShcF | 17.8 | 1.1E-11 |
| IYO_006850 | type III secretion system protein | 17.5 | 1.3E-13 |
| IYO_029040 | type III secretion chaperone CesT | 16.9 | 1.2E-12 |
| IYO_008282 | HopZ5 | 16.8 | 1.4E-10 |
| IYO_009200 | hypothetical protein | 16.8 | 4.7E-16 |
| IYO_005160 | HopI1 | 16.6 | 3.7E-21 |
| IYO_012125 | diguanylate cyclase | 16.1 | 2.4E-19 |
| IYO_006895 | type III secretion protein HrpI | 15.6 | 2.8E-30 |
| IYO_003600 | AvrB4 | 15.5 | 4.7E-22 |
| IYO_022140 | SAM-dependent methyltransferase | 14.6 | 3E-23 |
| IYO_012120 | AraC family transcriptional regulator | 13.9 | 9.3E-12 |
| IYO_002045 | hypothetical protein | 13.8 | 5.1E-05 |
| IYO_017375 | phosphonate/organophosphate ester transporter subunit | 13.2 | 9.7E-09 |
| IYO_018555 | HopAZ1 | 12.9 | 1.3E-16 |
| IYO_012115 | XRE family transcriptional regulator | 12.2 | 7.3E-21 |
| IYO_006745 | HopAA1-1 | 11.8 | 9.8E-12 |
| IYO_028535 | NADP transhydrogenase subunit alpha | 11.6 | 6.2E-09 |
| IYO_008285 | HopH1 | 11.4 | 1.3E-06 |
| IYO_008065 | AvrRpm1 | 11.4 | 5E-10 |
| IYO_010805 | LuxR family transcriptional regulator | 11.4 | 2.7E-09 |
| IYO_029795 | HopAU1 | 10.9 | 1.2E-05 |
| IYO_006770 | AvrE1 | 10.8 | 1.2E-21 |
| IYO_000845 | HopY1 | 10.7 | 1.2E-10 |
| IYO_003720 | HopAO2 | 10.7 | 7.7E-21 |
| IYO_012140 | protein tolQ | 10.2 | 4.9E-06 |
| IYO_022695 | alkaline phosphatase | 10.2 | 3.4E-13 |
| IYO_006250 | tail protein | 10.1 | 2.1E-20 |
| IYO_027360 | transcriptional initiation protein Tat | 9.7 | 1.7E-11 |
| IYO_009265 | serine/threonine protein phosphatase | 9.4 | 4.2E-08 |
| IYO_016255 | Ais protein | 9.2 | 3.8E-09 |
| IYO_024150 | HopR1 | 9.0 | 4.3E-25 |
| IYO_023400 | energy transducer TonB | 8.9 | 0.00019 |
| IYO_009660 | hypothetical protein | 8.9 | 0.00138 |
| IYO_027435 | DNA polymerase III subunit epsilon | 8.4 | 4E-08 |
| IYO_012610 | MarR family transcriptional regulator | 8.4 | 6.1E-05 |
| IYO_013150 | HopBN1 | 8.0 | 7.4E-12 |
| IYO_002040 | hypothetical protein | 8.0 | 0.00434 |
| IYO_012030 | nitrite reductase | 7.7 | 0.00011 |
| IYO_013145 | type III chaperone protein ShcF | 7.7 | 3.8E-07 |
| IYO_003727 | HopBB1-1 | 7.7 | 1.6E-08 |
| IYO_029288 | AvrRpm2 (frameshifts) | 7.5 | 2.3E-06 |
| IYO_012145 | biopolymer transporter TolR | 7.5 | 0.01418 |
| IYO_003315 | metal ABC transporter ATPase | 7.5 | 7.5E-14 |
| IYO_028380 | type III chaperone protein ShcA | 7.5 | 3.6E-07 |
| IYO_000385 | dodecin flavoprotein | 7.5 | 1.5E-06 |
| IYO_006845 | type III secretion system protein | 7.4 | 4.1E-10 |
| IYO_023505 | chemotaxis protein | 7.2 | 1.2E-07 |
| IYO_014240 | hypothetical | 7.2 | 4.7E-09 |
| IYO_028540 | NAD(P) transhydrogenase | 7.0 | 0.07674 |
| IYO_003680 | HopAF1 | 7.0 | 9.5E-10 |
| IYO_001870 | hypothetical protein | 6.8 | 1.8E-12 |
| IYO_005855 | UDP-N-acetylglucosamine 2-epimerase | 6.8 | 6.1E-13 |
| IYO_013690 | membrane protein | 6.7 | 2.4E-07 |
| IYO_010630 | thiamine biosynthesis protein ApbE | 6.6 | 7.4E-14 |
| IYO_011020 | chemotaxis protein | 6.4 | 7.1E-13 |
| IYO_023390 | biopolymer transporter ExbB | 6.4 | 0.0044 |
| IYO_029045 | HopZ3 | 6.3 | 1.6E-09 |
| IYO_024520 | voltage-gated chloride channel protein | 5.9 | 1.1E-05 |
| IYO_009335 | Fe-S oxidoreductase | 5.8 | 0.02136 |
| IYO_024535 | hypothetical protein | 5.8 | 0.00527 |
| IYO_004060 | hypothetical protein | 5.7 | 7.8E-27 |
| IYO_021665 | MFS transporter | 5.7 | 0.00017 |
| IYO_016185 | UDP-4-amino-4-deoxy-L-arabinose-oxoglutarate aminotransferase | 5.5 | 1.7E-05 |
| IYO_020420 | iron ABC transporter permease | 5.5 | 1.9E-16 |
| IYO_022135 | InaA protein | 5.5 | 3.1E-08 |
| IYO_007455 | membrane protein | 5.3 | 1.3E-13 |
| IYO_016195 | UDP-4-amino-4-deoxy-L-arabinose formyltransferase | 5.3 | 3.8E-08 |
| IYO_022030 | ACP phosphodiesterase | 5.2 | 5.5E-09 |
| IYO_006775 | lytic transglycosylase | 5.2 | 1.4E-13 |
| IYO_012605 | fusaric acid resistance protein | 5.2 | 4.7E-05 |
| IYO_018725 | membrane protein | 5.1 | 0.00024 |
| IYO_004240 | hypothetical protein | 5.0 | 6E-05 |
| IYO_014250 | chemotaxis protein CheY | 5.0 | 0.10469 |
