# Supplementary material for: Re-programming of Pseudomonas syringae pv. actinidiae gene expression during early stages of infection of kiwifruit
Source: BMC Genomics. 2018 Nov 15;19:822. doi: 10.1186/s12864-018-5197-5 (PMC6238374; doi:10.1186/s12864-018-5197-5)
Supplement: Supplementary file 5 — Expression levels of individual Psa effectors over the infection time course. Effectors are ranked by the highest level of expression between 3 and 12 h post infection (HPI) in reads per kilobase per million (RPKM). Effectors likely to be disrupted, duplicated or pseudogenes were not included. (DOCX 22 kb) [file 12864_2018_5197_MOESM5_ESM.docx]

Additional file 5. Expression levels of individual *Psa* effectors over the infection time course. Effectors are ranked by the highest level of expression between 3 and 12 hours post inoculation (HPI) in Reads Per Kilobase per Million (RPKM). Effectors likely to be disrupted or pseudogenes were not included. ND = not determined.

|  |  |  | RPKM | | | | | | | | | |
| --- | --- | --- | --- | --- | --- | --- | --- | --- | --- | --- | --- | --- |
| Gene ID | Effector | Heat map group | *in vitro* | 1.5 HPI | 3 HPI | 6 HPI | 12 HPI | 24 HPI | 48 HPI | 72 HPI | 96 HPI | 120 HPI |
| IYO_029795 | HopAU1 | 11 | 346.4 | 970.6 | 3772.5 | 2876.6 | 3196.1 | 2141.7 | 1138.8 | 952.1 | 619.7 | 946.2 |
| IYO_008065 | AvrRpm1 | 11 | 123.9 | 365.5 | 1293.2 | 867.8 | 1415.6 | 940.2 | 538.8 | 542.9 | 526.2 | 693.6 |
| IYO_020425 | AvrPto5 | 11 | 58.8 | 331 | 1198.8 | 920.8 | 1368.6 | 770.7 | 472.4 | 424.3 | 281.7 | 487.2 |
| IYO_018555 | HopAZ1 | 11 | 102.1 | 226.8 | 995 | 616.3 | 1320.1 | 1178.3 | 479.8 | 682.5 | 464.8 | 788 |
| IYO_004052 | HopS2 | 11 | 15 | 181.7 | 1145.3 | 751.5 | 762.2 | 494.6 | 324.2 | 239.9 | 129 | 226.5 |
| IYO_003720 | HopAO2 | 11 | 102.7 | 346.6 | 1102.7 | 664.4 | 821.5 | 560.7 | 357.2 | 378.4 | 228.5 | 356.7 |
| IYO_003600 | AvrB4 | 11 | 65.6 | 326.8 | 925.5 | 696.7 | 1015.7 | 621.4 | 332.6 | 431.6 | 251.2 | 465.3 |
| IYO_013150 | HopBN1 | 11 | 105.3 | 179.4 | 633 | 468.2 | 843.7 | 622.5 | 327.7 | 440.3 | 232.6 | 342.3 |
| IYO_008282 | HopZ5 | 11 | 43.4 | 314.1 | 662.1 | 546.9 | 727.8 | 426.2 | 222 | 186.1 | 141.9 | 440.9 |
| IYO_003570 | avrD1 | 6 | 38.2 | 99.4 | 432.1 | 412.6 | 687.1 | 492.6 | 240.1 | 419.9 | 264.3 | 703.8 |
| IYO_024217 | HopF2 | 11 | 187.9 | 225.4 | 658.8 | 479.4 | 649.8 | 563.5 | 288 | 400.6 | 213.4 | 403 |
| IYO_003657 | HopAW1 | 11 | 195 | 166.1 | 401.6 | 375.5 | 539 | 402.8 | 208.9 | 254.2 | 187 | 361.9 |
| IYO_006735 | HopN1 | 7 | 200.4 | 301.6 | 380.7 | 510.9 | 478.6 | 467.8 | 383.2 | 394.1 | 347.3 | 314.1 |
| IYO_008285 | HopH1 | 11 | 43.1 | 147.6 | 493.5 | 241.2 | 462.3 | 284.1 | 135.7 | 158.6 | 80.9 | 177.6 |
| IYO_000845 | HopY1 | 11 | 42.8 | 121.7 | 426.6 | 279.2 | 459.5 | 317 | 172.9 | 238.8 | 115.9 | 204.8 |
| IYO_005160 | HopI1 | 11 | 22.3 | 96.9 | 370 | 246.3 | 367 | 324.7 | 169.6 | 217.6 | 143.6 | 181.2 |
| IYO_006760 | HopM1 | 11 | 20.3 | 106.4 | 368.9 | 286.3 | 347.5 | 186.1 | 98.1 | 102.9 | 69.2 | 114.3 |
| IYO_003727 | HopBB1-1 | 11 | 47.3 | 103.6 | 362.9 | 266.3 | 232.3 | 173.2 | 76.2 | 107 | 143.6 | 100.6 |
| IYO_003680 | HopAF1 | 6 | 46 | 90.6 | 176.3 | 145.4 | 302.4 | 321.1 | 145.9 | 186.5 | 154.3 | 213.1 |
| IYO_029045 | HopZ3 | 11 | 47.3 | 89.7 | 248.7 | 179.1 | 300.1 | 206.6 | 111.2 | 116.5 | 84.1 | 135.8 |
| IYO_006770 | AvrE1 | 11 | 27 | 73.9 | 291.1 | 176.3 | 164.6 | 129.1 | 57.6 | 53.6 | 44.9 | 47.8 |
| IYO_003525 | HopQ1 | 11 | 53.6 | 96.9 | 165 | 163.3 | 232.5 | 146.8 | 86.5 | 131.6 | 79.6 | 168.7 |
| IYO_003530 | HopD1 | 7 | 53.5 | 99.3 | 212.3 | 184 | 229.5 | 216.6 | 156.4 | 170.4 | 124.3 | 156.3 |
| IYO_024150 | HopR1 | 11 | 24.1 | 58.5 | 217.6 | 103 | 94 | 68.1 | 77.2 | 112.8 | 45.6 | 94.9 |
| IYO_012225 | HopAE1 | 11 | 31.2 | 53.1 | 133.8 | 95.1 | 142.7 | 174.9 | 115.4 | 111.7 | 71.9 | 121.5 |
| IYO_003675 | HopBB1-2 | 6 | 32.1 | 40.7 | 129.6 | 67.4 | 148.5 | 104.5 | 55.6 | 50.1 | 21.9 | 136.4 |
| IYO_027420 | HopAS1 | 9 | 24.9 | 11.4 | 12.3 | 17.1 | 21.7 | 60.3 | 129.9 | 109.4 | 38.4 | 53.4 |
| IYO_023985 | HopAH1 | 1 | 53.5 | 111.4 | 111.3 | 110.1 | 81.9 | 93.5 | 70.5 | 82.1 | 57.6 | 42.3 |
| IYO_006745 | HopAA1-1 | 11 | 9.2 | 27.7 | 107.2 | 60.6 | 109.3 | 80.5 | 32.5 | 27.7 | 12.8 | 31 |
| IYO_003635 | HopX3* | 5 | 53.1 | 93.4 | 67.9 | 70.6 | 65.6 | 33.7 | 63.6 | 107.1 | 59.7 | 88.2 |
| IYO_023205 | HopAM1-1 | ND |  |  |  |  |  |  |  |  |  |  |
| IYO_008385 | HopAM1-2 | ND |  |  |  |  |  |  |  |  |  |  |
