# Supplementary material for: Re-programming of Pseudomonas syringae pv. actinidiae gene expression during early stages of infection of kiwifruit
Source: BMC Genomics. 2018 Nov 15;19:822. doi: 10.1186/s12864-018-5197-5 (PMC6238374; doi:10.1186/s12864-018-5197-5)
Supplement: Supplementary file 6 — Expression of non-effector genes with upstream HrpL boxes. Genes are ranked by the ration of expression at 12 h post infection (HPI) compared with in vitro expression. (DOCX 18 kb) [file 12864_2018_5197_MOESM6_ESM.docx]

Additional file 5. Expression of non-effector genes with *hrp* boxes. Genes are ranked by the ration of expression at 12 hours post infection (HPI) compared with *in vitro* expression.

| Gene ID | Gene Annotation | Cluster | 12 HPI/*in vitro* | *P*-value |
| --- | --- | --- | --- | --- |
| IYO_002060 | IAA lysine ligase | 11 | 36.0 | 6.20E-17 |
| IYO_006775 | lytic transglycosylase | 11 | 5.2 | 5.56E-11 |
| IYO_010630 | thiamine biosynthesis protein ApbE | 11 | 5 | 5.79E-14 |
| IYO_027210 | peptidase M20 | 11 | 3.5 | 1.16E-05 |
| IYO_025425 | phosphatidylserine decarboxylase | 11 | 3.4 | 1.27E-01 |
| IYO_002055 | multidrug transporter Mate | 7 | 2.5 | 1.42E-02 |
| IYO_000225 | AraC transcription factor | 8 | 1.5 | 2.89E-01 |
| IYO_008215 | Transporter | 1 | 1.2 | 3.33E-01 |
