# Supplementary material for: Re-programming of Pseudomonas syringae pv. actinidiae gene expression during early stages of infection of kiwifruit
Source: BMC Genomics. 2018 Nov 15;19:822. doi: 10.1186/s12864-018-5197-5 (PMC6238374; doi:10.1186/s12864-018-5197-5)
Supplement: Supplementary file 7 — Genes expressed in the mid phase of infection that do not encode the T3SS or T3SEs. Effectors were ranked by the highest level of expression between 3 and 24 HPI compared with in vitro (cutoff 5-fold). (DOCX 21 kb) [file 12864_2018_5197_MOESM7_ESM.docx]

Additional file 7. Genes expressed in the mid phase of infection that do not encode T3SS or T3SEs. Genes were ranked by the highest level of expression between 3 and 24 hours post inoculation compared with *in vitro* (cutoff 5-fold).

| Gene ID | Gene Annotation | Max 3-24hr/*in vitro* | *P*-value |
| --- | --- | --- | --- |
| IYO_022020 | hemolysin | 176.8965134 | 9.7E-44 |
| IYO_012110 | Ais protein | 52.15177309 | 6.6E-21 |
| IYO_022025 | glycerol acyltransferase | 47.41414785 | 4E-16 |
| IYO_002060 | IAA lysine ligase | 46.43964929 | 6.2E-20 |
| IYO_006830 | secretin | 44.12981547 | 1E-37 |
| IYO_028770 | LysR family transcriptional regulator | 44.1000272 | 4.7E-25 |
| IYO_003325 | copper resistance protein CopZ | 41.36623134 | 8.7E-27 |
| IYO_012005 | phosphate ABC transporter permease | 37.70442846 | 3.1E-32 |
| IYO_028960 | sulfonate ABC transporter permease | 35.71674044 | 1.4E-12 |
| IYO_006420 | chemotaxis protein | 35.6663794 | 4.3E-25 |
| IYO_014395 | lytic transglycosylase | 31.15779288 | 4.2E-24 |
| IYO_014235 | hypothetical protein | 30.42819287 | 4.3E-21 |
| IYO_028955 | nitrate ABC transporter ATP-binding protein | 28.30708753 | 4.5E-08 |
| IYO_005735 | glycoside hydrolase | 25.83644908 | 1.2E-24 |
| IYO_006885 | ATP synthase | 21.27236361 | 1.6E-21 |
| IYO_014245 | membrane protein | 17.92814971 | 0.01651 |
| IYO_009200 | hypothetical protein | 16.75685698 | 4.7E-16 |
| IYO_012125 | diguanylate cyclase | 16.10227356 | 2.4E-19 |
| IYO_022140 | SAM-dependent methyltransferase | 14.56526101 | 3E-23 |
| IYO_012120 | AraC family transcriptional regulator | 13.86039793 | 9.3E-12 |
| IYO_002045 | hypothetical protein | 13.78948606 | 5.1E-05 |
| IYO_017375 | phosphonate/organophosphate ester transporter subunit | 13.19762412 | 9.7E-09 |
| IYO_012115 | XRE family transcriptional regulator | 12.15821469 | 7.3E-21 |
| IYO_028535 | NADP transhydrogenase subunit alpha | 11.6258924 | 6.2E-09 |
| IYO_010805 | LuxR family transcriptional regulator | 11.35613537 | 2.7E-09 |
| IYO_012140 | protein tolQ | 10.24591846 | 2.9E-05 |
| IYO_022695 | alkaline phosphatase | 10.1825476 | 3.4E-13 |
| IYO_006250 | tail protein | 10.12427557 | 2.1E-20 |
| IYO_027360 | transcriptional initiation protein Tat | 9.73508113 | 1.7E-11 |
| IYO_009265 | serine/threonine protein phosphatase | 9.394839603 | 4.2E-08 |
| IYO_016255 | Ais protein | 9.226696251 | 3.8E-09 |
| IYO_023400 | energy transducer TonB | 8.931602714 | 0.00019 |
| IYO_009660 | hypothetical protein | 8.858717065 | 0.00138 |
| IYO_027435 | DNA polymerase III subunit epsilon | 8.414824567 | 4E-08 |
| IYO_012610 | MarR family transcriptional regulator | 8.394245239 | 6.1E-05 |
| IYO_002040 | hypothetical protein | 7.97028053 | 0.00434 |
| IYO_012030 | nitrite reductase | 7.725691653 | 0.00011 |
| IYO_012145 | biopolymer transporter TolR | 7.522804764 | 0.01832 |
| IYO_003315 | metal ABC transporter ATPase | 7.519255209 | 7.5E-14 |
| IYO_000385 | dodecin flavoprotein | 7.463415413 | 1.5E-06 |
| IYO_023505 | chemotaxis protein | 7.205132932 | 1.2E-07 |
| IYO_014240 | hypothetical | 7.163653846 | 4.7E-09 |
| IYO_028540 | NAD(P) transhydrogenase | 6.987308302 | 0.07674 |
| IYO_001870 | hypothetical protein | 6.828648569 | 1.8E-12 |
| IYO_005855 | UDP-N-acetylglucosamine 2-epimerase | 6.828589164 | 6.1E-13 |
| IYO_013690 | membrane protein | 6.663626577 | 2.4E-07 |
| IYO_010630 | thiamine biosynthesis protein ApbE | 6.594972584 | 7.4E-14 |
| IYO_011020 | chemotaxis protein | 6.419561085 | 7.1E-13 |
| IYO_023390 | biopolymer transporter ExbB | 6.37203283 | 0.0044 |
| IYO_024520 | voltage-gated chloride channel protein | 5.859449978 | 1.1E-05 |
| IYO_009335 | Fe-S oxidoreductase | 5.832304804 | 0.01462 |
| IYO_024535 | hypothetical protein | 5.789314032 | 0.00527 |
| IYO_004060 | hypothetical protein | 5.695280181 | 7.8E-27 |
| IYO_021665 | MFS transporter | 5.684151414 | 0.00017 |
| IYO_016185 | UDP-4-amino-4-deoxy-L-arabinose-oxoglutarate aminotransferase | 5.505631007 | 1.7E-05 |
| IYO_020420 | iron ABC transporter permease | 5.488234589 | 1.9E-16 |
| IYO_022135 | InaA protein | 5.460963656 | 3.1E-08 |
| IYO_007455 | membrane protein | 5.347108089 | 1.3E-13 |
| IYO_016195 | UDP-4-amino-4-deoxy-L-arabinose formyltransferase | 5.310883424 | 3.8E-08 |
| IYO_022030 | ACP phosphodiesterase | 5.183953282 | 5.5E-09 |
| IYO_006775 | lytic transglycosylase | 5.161048509 | 1.4E-13 |
| IYO_012605 | fusaric acid resistance protein | 5.150295806 | 4.7E-05 |
| IYO_018725 | membrane protein | 5.138633214 | 0.00024 |
| IYO_004240 | hypothetical protein | 4.992962804 | 6E-05 |
| IYO_014250 | chemotaxis protein CheY | 4.96231316 | 0.10469 |
