# Supplementary material for: Re-programming of Pseudomonas syringae pv. actinidiae gene expression during early stages of infection of kiwifruit
Source: BMC Genomics. 2018 Nov 15;19:822. doi: 10.1186/s12864-018-5197-5 (PMC6238374; doi:10.1186/s12864-018-5197-5)
Supplement: Supplementary file 8 — Genes upregulated late in the time course . Genes were ranked based on the ratio of expression at 120 h post infection (HPI) compared with 1.5 HPI. (DOCX 22 kb) [file 12864_2018_5197_MOESM8_ESM.docx]

Additional file 8. Late upregulated genes. Genes were ranked based on the ratio of expression at 120 hours post inoculation (HPI) compared with 1.5 HPI. RPKM, reads per kilobase per million.

| Gene ID | Gene Annotation | 120HPI RPKM/1.5 HPI RPKM | *P*-value |
| --- | --- | --- | --- |
| IYO_016130 | ABC transporter | 119.9 | 6.92E-05 |
| IYO_016135 | acyl-CoA synthetase | 93.1 | 7.11E-06 |
| IYO_006065 | glycosyl transferase | 75.6 | 4.34E-08 |
| IYO_013825 | alkanesulfonate monooxygenase | 51.2 | 4.06E-15 |
| IYO_006070 | GDP-mannose dehydrogenase | 51.1 | 7.92E-40 |
| IYO_009605 | YqcI/YcgG family protein | 39.1 | 8.72E-07 |
| IYO_023405 | biopolymer transporter ExbD | 34.3 | 6.02E-05 |
| IYO_026605 | monooxygenase | 33.2 | 2.77E-11 |
| IYO_017250 | energy transducer TonB | 28.3 | 2.71E-08 |
| IYO_017245 | biopolymer transporter ExbB | 28.0 | 5.43E-07 |
| IYO_027905 | transporter | 26.9 | 3.73E-10 |
| IYO_016105 | acyl-CoA dehydrogenase | 24.8 | 1.23E-07 |
| IYO_005580 | hypothetical protein | 24.2 | 2.93E-12 |
| IYO_027910 | aliphatic sulfonates transport ATP-binding subunit | 23.7 | 5.36E-06 |
| IYO_006055 | alginate biosynthesis protein | 22.7 | 2.51E-06 |
| IYO_006050 | alginate regulatory protein | 20.0 | 6.29E-08 |
| IYO_009255 | sulfonate ABC transporter ATP-binding protein | 19.9 | 8.75E-03 |
| IYO_006015 | mannose-1-phosphate guanylyltransferase | 19.3 | 1.91E-22 |
| IYO_018210 | calcium-binding protein | 19.2 | 2.34E-16 |
| IYO_008315 | lipoprotein | 18.3 | 8.74E-18 |
| IYO_016100 | acyl-CoA dehydrogenase | 18.2 | 1.37E-03 |
| IYO_016120 | ABC transporter permease | 16.7 | 7.26E-03 |
| IYO_026615 | N5,N10-methylene tetrahydromethanopterin reductase | 16.5 | 3.86E-07 |
| IYO_015950 | polar amino acid ABC transporter permease | 15.6 | 3.90E-02 |
| IYO_006060 | hemolysin D | 15.3 | 5.79E-11 |
| IYO_006040 | alginate O-acetyltransferase | 15.1 | 1.94E-09 |
| IYO_026620 | methionine ABC transporter substrate-binding protein | 15.0 | 1.95E-03 |
| IYO_026625 | ABC transporter | 15.0 | 6.50E-07 |
| IYO_027920 | ABC transporter substrate-binding protein | 14.1 | 2.03E-09 |
| IYO_015300 | ABC transporter permease | 13.1 | 3.01E-03 |
| IYO_026675 | sulfonate ABC transporter ATP-binding protein | 12.7 | 2.93E-04 |
| IYO_006030 | poly(beta-D-mannuronate) O-acetylase | 12.3 | 2.14E-09 |
| IYO_011010 | catalase | 12.0 | 4.25E-14 |
| IYO_016095 | 5,10-methylene tetrahydromethanopterin reductase | 11.7 | 2.57E-07 |
| IYO_014785 | sugar ABC transporter | 11.4 | 1.52E-06 |
| IYO_026610 | acyl-CoA dehydrogenase | 11.0 | 1.56E-05 |
| IYO_006025 | alginate O-acetyltransferase | 11.0 | 1.43E-07 |
| IYO_012440 | hypothetical protein | 10.7 | 1.66E-02 |
| IYO_020560 | peptidase M4 | 10.4 | 3.46E-09 |
| IYO_011310 | NAD(P)H-dependent FMN reductase | 10.2 | 9.59E-06 |
| IYO_024090 | porin | 10.1 | 1.15E-08 |
| IYO_003290 | hypothetical protein | 10.0 | 9.64E-02 |
| IYO_010385 | lipoprotein | 10.0 | 1.85E-02 |
| IYO_027985 | hypothetical protein | 9.8 | 1.90E-07 |
| IYO_006045 | poly(beta-D-mannuronate) C5 epimerase | 9.6 | 1.11E-09 |
| IYO_006035 | poly(beta-D-mannuronate) lyase | 9.6 | 4.76E-11 |
| IYO_014780 | sugar ABC transporter substrate-binding protein | 9.5 | 1.68E-06 |
| IYO_016140 | monooxygenase | 9.1 | 1.75E-06 |
| IYO_017240 | biopolymer transporter ExbD | 9.1 | 1.69E-05 |
| IYO_011305 | lysine transporter LysE | 8.7 | 8.38E-05 |
| IYO_001790 | taurine transporter ATP-binding subunit | 8.7 | 6.15E-02 |
| IYO_026775 | alpha/beta hydrolase | 8.6 | 1.81E-07 |
| IYO_024095 | ABC transporter substrate-binding protein | 8.5 | 6.24E-08 |
| IYO_027980 | ABC transporter permease | 8.1 | 6.65E-12 |
| IYO_026630 | ABC transporter permease | 7.8 | 2.58E-02 |
| IYO_001460 | prophage PssSM-01 | 7.7 | 2.51E-03 |
| IYO_011840 | hemolysin D | 7.7 | 2.28E-03 |
| IYO_013055 | aldolase | 7.4 | 1.26E-04 |
| IYO_026680 | taurine dioxygenase | 7.2 | 9.94E-08 |
| IYO_014465 | hypothetical protein | 6.9 | 1.11E-09 |
| IYO_014710 | lipoprotein | 6.8 | 7.90E-09 |
| IYO_017510 | lipoprotein | 6.8 | 1.75E-01 |
| IYO_001820 | hypothetical protein | 6.8 | 4.01E-02 |
| IYO_027915 | alkanesulfonate transporter permease subunit | 6.7 | 2.33E-04 |
| IYO_016110 | branched-chain amino acid ABC transporter ATP-binding protein | 6.6 | 4.90E-03 |
| IYO_009290 | LTXXQ domain-containing protein | 6.6 | 1.85E-15 |
| IYO_006935 | hypothetical protein | 6.5 | 2.86E-08 |
| IYO_016115 | ABC transporter permease | 6.3 | 8.18E-05 |
| IYO_009230 | sulfurtransferase | 6.1 | 2.96E-04 |
| IYO_013050 | nitrate ABC transporter substrate-binding protein | 6.0 | 2.86E-02 |
| IYO_020620 | hypothetical protein | 5.8 | 2.46E-05 |
| IYO_011220 | Fis family transcriptional regulator | 5.8 | 2.93E-06 |
| IYO_029660 | coenzyme F390 synthetase (plasmid) | 5.6 | 1.54E-01 |
| IYO_027965 | sulfate ABC transporter ATP-binding protein | 5.4 | 7.96E-04 |
| IYO_005875 | hypothetical protein | 5.4 | 7.26E-14 |
| IYO_016125 | ABC transporter permease | 5.4 | 7.52E-02 |
| IYO_001465 | prophage PssSM-01 | 5.3 | 1.02E-04 |
| IYO_011375 | class V aminotransferase | 5.3 | 4.08E-06 |
| IYO_009250 | ABC transporter permease | 5.3 | 1.92E-01 |
| IYO_001810 | ribonucleotide reductase | 5.2 | 1.16E-14 |
| IYO_004495 | hypothetical protein | 5.1 | 9.88E-03 |
| IYO_001805 | transposase | 5.1 | 3.42E-06 |
| IYO_028000 | diguanylate cyclase | 5.0 | 3.01E-06 |
| IYO_009615 | serine dehydratase | 5.0 | 1.85E-07 |
