# Supplementary material for: Re-programming of Pseudomonas syringae pv. actinidiae gene expression during early stages of infection of kiwifruit
Source: BMC Genomics. 2018 Nov 15;19:822. doi: 10.1186/s12864-018-5197-5 (PMC6238374; doi:10.1186/s12864-018-5197-5)
Supplement: Supplementary file 9 — Expression levels of secondary metabolite gene clusters. Means of reads per kilobase per million (RPKM) for each gene across all time points with standard deviations. (DOCX 13 kb) [file 12864_2018_5197_MOESM9_ESM.docx]

Additional file 9. Expression levels of secondary metabolite gene clusters. Reads Per Kilobase per Million (RPKM) were means plus/minus standard deviation for each gene across all time points.

| Secondary metabolite pathway | Function | Gene members | Induction levels *in planta* |
| --- | --- | --- | --- |
| Novel Non-ribosomal peptide synthetase | unknown | IYO_003775-003830 | Constitutive expression *in planta* (average RPKM 67 +/- 43) |
| Pyoverdine | Iron chelation | IYO_010820-010910 | Constitutive expression *in planta* (average RPKM 41 +/- 43) |
| Achromobactin | Iron chelation | IYO_013460-013515 | Constitutive expression *in planta* (average RPKM 17 +/- 9) |
| Yersiniabactin | Iron chelation | IYO_013840-013910 | Constitutive expression *in planta* (average RPKM 15 +/-19) |
| Unknown | unknown | IYO_026725-026760 | Weak constitutive *in planta* (expression 48 +/-16 RPKM) |
| Mangotoxin | Inhibitor of ornithine deacetylase | IYO_028470-028715 | Weak expressed *in planta* (average RPKM 13 +/- 9) |
| Plasmid-borne pathway | unknown | IYO_029645-029685 | Induced late *in planta* (see figure 5) |
