# Supplementary material for: Re-programming of Pseudomonas syringae pv. actinidiae gene expression during early stages of infection of kiwifruit
Source: BMC Genomics. 2018 Nov 15;19:822. doi: 10.1186/s12864-018-5197-5 (PMC6238374; doi:10.1186/s12864-018-5197-5)
Supplement: Supplementary file 10 — Reads per kilobase per million (RPKM) values of genes encoding proteins predicted to be secreted via T2SS. (DOCX 25 kb) [file 12864_2018_5197_MOESM10_ESM.docx]

Additional file 10. Reads per Kilobase per million values of genes encoding proteins predicted to be secreted via T2SS. HPI, hours post infection.

| # ID | Function | Phase | *in vitro* | 1.5 HPI | 3 HPI | 12 HPI | 24 HPI | 8 HPI | 72 HPI | 96 HPI | 120 HPI |
| --- | --- | --- | --- | --- | --- | --- | --- | --- | --- | --- | --- |
| IYO_011995 | phosphate ABC transporter substrate-binding protein | Early | 39.5 | 3366.6 | 5876.1 | 3269.3 | 2774.8 | 1220.1 | 1143.4 | 1114.5 | 1774.3 |
| IYO_019585 | thioredoxin | Early | 59.4 | 1525.9 | 1863.2 | 983.2 | 433.9 | 374.4 | 329.6 | 350.7 | 469.3 |
| IYO_027385 | ABC transporter substrate-binding protein | Early | 10.4 | 222.5 | 306.4 | 105.9 | 117.6 | 54.4 | 68.2 | 7.9 | 58.9 |
| IYO_006115 | amino acid ABC transporter substrate-binding protein | Early | 117.7 | 3408.6 | 2742.5 | 843.8 | 829.8 | 596.2 | 750.2 | 622.4 | 722.3 |
| IYO_028665 | phosphate-binding protein | Early | 33.6 | 759.5 | 683.8 | 278.3 | 194.3 | 145.9 | 138.5 | 166.4 | 210.9 |
| IYO_000970 | ammonia channel protein | Early | 43.5 | 1336.6 | 811.4 | 274.2 | 268.1 | 231.2 | 409.6 | 259.8 | 426.5 |
| IYO_021410 | short-chain dehydrogenase | Early | 6.6 | 142.6 | 113.3 | 26.3 | 33.4 | 19.3 | 4.4 | 19.6 | 12.1 |
| IYO_010675 | phosphatase | Early | 32.6 | 228.2 | 410.1 | 158.8 | 97.9 | 151.1 | 56.9 | 80.3 | 89.7 |
| IYO_021050 | ABC transporter substrate-binding protein | Early | 55.0 | 945.8 | 685.8 | 281.4 | 281.5 | 212.0 | 245.3 | 245.3 | 224.0 |
| IYO_020035 | ABC transporter substrate-binding protein | Early | 131.3 | 1169.0 | 1415.7 | 583.5 | 416.4 | 253.4 | 327.7 | 249.7 | 292.0 |
| IYO_014740 | sugar ABC transporter substrate-binding protein | Early | 59.2 | 490.2 | 604.3 | 243.3 | 163.4 | 143.6 | 89.5 | 131.6 | 96.6 |
| IYO_020310 | hypothetical protein | Early | 24.9 | 380.2 | 242.5 | 139.5 | 139.0 | 187.8 | 112.7 | 43.1 | 75.4 |
| IYO_004585 | branched-chain amino acid ABC transporter substrate-binding protein | Early | 87.8 | 956.2 | 489.1 | 174.2 | 232.2 | 130.6 | 255.0 | 172.8 | 338.3 |
| IYO_025190 | protein hupE | Early | 25.0 | 198.5 | 123.6 | 36.9 | 61.1 | 38.8 | 90.6 | 89.1 | 123.2 |
| IYO_006385 | porin | Early | 1383.7 | 7387.9 | 6833.1 | 1618.0 | 1721.0 | 1130.5 | 1055.6 | 984.5 | 945.0 |
| IYO_020485 | glycine/betaine ABC transporter substrate-binding protein | Early | 33.5 | 134.6 | 129.0 | 35.6 | 46.6 | 43.4 | 44.4 | 37.1 | 32.5 |
| IYO_008325 | polygalacturonase | Early | 164.0 | 369.3 | 564.7 | 150.2 | 140.3 | 107.1 | 97.9 | 83.8 | 107.4 |
| IYO_021455 | Methylamine utilization protein MauL | Early | 94.4 | 582.6 | 317.5 | 319.1 | 399.1 | 422.6 | 247.9 | 279.5 | 147.7 |
| IYO_004580 | urea ABC transporter permease | Early | 14.9 | 74.3 | 43.4 | 20.5 | 16.3 | 35.5 | 30.8 | 19.4 | 22.0 |
| IYO_026915 | amino acid ABC transporter substrate-binding protein | Early | 50.4 | 426.4 | 139.6 | 38.5 | 65.7 | 44.0 | 87.4 | 35.9 | 98.4 |
| IYO_006365 | sugar ABC transporter substrate-binding protein | Early | 1316.7 | 2310.1 | 3559.7 | 671.4 | 762.4 | 398.4 | 474.9 | 375.7 | 335.4 |
| IYO_024670 | hypothetical protein part of ICE | Early | 34.2 | 23.9 | 18.9 | 59.0 | 31.6 | 29.2 | 22.7 | 8.5 | 38.9 |
| IYO_006805 | type III secretion protein | Mid | 57.1 | 309.6 | 1263.6 | 1201.0 | 751.4 | 414.5 | 501.0 | 276.3 | 450.1 |
| IYO_002045 | hypothetical protein | Mid | 73.0 | 63.8 | 30.4 | 1006.5 | 102.8 | 120.8 | 106.7 | 104.0 | 50.3 |
| IYO_009660 | hypothetical protein | Mid | 31.6 | 11.5 | 0.0 | 280.3 | 84.6 | 121.0 | 73.3 | 66.4 | 81.0 |
| IYO_002040 | hypothetical protein | Mid | 60.1 | 51.1 | 27.5 | 479.1 | 77.9 | 74.5 | 104.8 | 106.2 | 117.1 |
| IYO_001870 | hypothetical protein | Mid | 510.6 | 318.1 | 478.1 | 2793.5 | 2242.8 | 1524.5 | 1518.2 | 1974.0 | 1162.6 |
| IYO_006020 | alginate O-acetyltransferase | Mid | 88.2 | 49.0 | 27.9 | 423.9 | 197.5 | 278.0 | 383.9 | 259.6 | 417.2 |
| IYO_023395 | TonB-dependent receptor | Mid | 25.2 | 19.7 | 20.4 | 98.7 | 101.4 | 46.8 | 37.8 | 47.0 | 83.2 |
| IYO_008760 | sorbosone dehydrogenase | Mid | 105.4 | 49.9 | 40.3 | 391.5 | 269.6 | 218.2 | 157.1 | 176.8 | 125.4 |
| IYO_018720 | sorbosone dehydrogenase | Mid | 60.3 | 37.9 | 25.8 | 224.0 | 111.0 | 97.4 | 102.4 | 122.0 | 93.4 |
| IYO_027210 | peptidase M20 | Mid | 53.5 | 35.4 | 114.4 | 189.8 | 132.1 | 98.8 | 88.0 | 68.1 | 119.3 |
| IYO_022715 | phospholipid-binding protein | Mid | 8230.8 | 2550.5 | 2568.7 | 29098.2 | 18992.7 | 15528.5 | 13209.7 | 16287.6 | 10178.4 |
| IYO_004060 | hypothetical protein | Mid | 1959.4 | 7898.0 | 5063.3 | 6887.1 | 5478.9 | 4687.6 | 4501.3 | 3573.6 | 3898.1 |
| IYO_019200 | BNR/Asp-box repeat-containing protein | Mid | 56.0 | 117.1 | 157.2 | 196.2 | 133.8 | 88.1 | 59.6 | 80.2 | 66.9 |
| IYO_004055 | membrane protein | Mid | 34.6 | 39.5 | 114.7 | 86.9 | 62.9 | 37.4 | 39.6 | 39.0 | 33.7 |
| IYO_006835 | type III secretion protein | Mid | 312.2 | 275.5 | 532.0 | 847.3 | 672.8 | 399.1 | 381.4 | 330.7 | 383.1 |
| IYO_020600 | ABC transporter substrate-binding protein | Mid | 76.1 | 47.3 | 60.0 | 198.5 | 143.9 | 138.9 | 98.6 | 106.3 | 119.6 |
| IYO_022515 | toluene tolerance protein | Mid | 116.7 | 73.1 | 109.9 | 251.8 | 129.2 | 148.1 | 174.9 | 121.3 | 128.6 |
| IYO_006575 | superoxide dismutase | Mid | 178.1 | 46.6 | 35.6 | 375.8 | 202.9 | 114.6 | 173.8 | 176.6 | 223.3 |
| IYO_022005 | hypothetical protein | Mid | 45.1 | 33.6 | 34.6 | 88.2 | 69.7 | 55.2 | 0.0 | 17.9 | 66.5 |
| IYO_027595 | phosphorylcholine phosphatase | Mid | 50.4 | 33.8 | 36.3 | 82.4 | 97.7 | 53.8 | 62.8 | 63.3 | 64.2 |
| IYO_011990 | hypothetical protein | Mid | 1900.4 | 539.1 | 489.4 | 3491.9 | 1831.7 | 1693.3 | 1220.1 | 1023.3 | 836.5 |
| IYO_027840 | ABC transporter substrate-binding protein | Mid | 70.8 | 93.0 | 65.3 | 56.8 | 99.4 | 36.2 | 24.1 | 37.9 | 42.6 |
| IYO_017485 | hypothetical protein | Mid | 300.2 | 114.9 | 115.4 | 411.4 | 188.4 | 259.0 | 298.3 | 216.1 | 202.3 |
| IYO_010560 | cytochrome C | Mid | 86.6 | 54.4 | 56.6 | 92.6 | 76.9 | 66.5 | 80.4 | 56.4 | 49.9 |
| IYO_014770 | hypothetical protein | Mid | 208.1 | 169.6 | 157.6 | 200.6 | 190.5 | 206.4 | 197.7 | 172.8 | 410.9 |
| IYO_011885 | type III effector | Mid | 25.2 | 6.4 | 3.6 | 7.2 | 7.8 | 8.5 | 6.0 | 3.4 | 2.9 |
