# Supplementary material for: Re-programming of Pseudomonas syringae pv. actinidiae gene expression during early stages of infection of kiwifruit
Source: BMC Genomics. 2018 Nov 15;19:822. doi: 10.1186/s12864-018-5197-5 (PMC6238374; doi:10.1186/s12864-018-5197-5)
Supplement: Supplementary file 12 — RNA-seq experimental design. Three pottles, each with three plantlets, were inoculated for each time point. For RNA extraction, one plantlet from each pottle, was harvested and combined for each of three biological replicates. (DOCX 229 kb) [file 12864_2018_5197_MOESM12_ESM.docx]

Additional file 12. RNA-seq experimental design
